# Influence of solvent mixture on nucleophilicity parameters: the case of pyrrolidine in methanol–acetonitrile†

**DOI:** 10.1039/d0ra06324j

**Published:** 2020-08-03

**Authors:** Salma Souissi, Wahiba Gabsi, Abderraouf Echaieb, Julien Roger, Jean-Cyrille Hierso, Paul Fleurat-Lessard, Taoufik Boubaker

**Affiliations:** Université de Monastir, Faculté des Sciences, Laboratoire de Chimie Hétérocyclique, Produits Naturels et Réactivité (LR11S39) Avenue de l’Environnement 5019 Monastir Tunisia boubaker_taoufik@yahoo.fr; Institut de Chimie Moléculaire de l’Université de Bourgogne (UMR-CNRS 6302), Université Bourgogne Franche-Comté (UBFC) 9 Avenue Alain Savary 21078 Dijon France Paul.Fleurat-Lessard@u-bourgogne.fr

## Abstract

The course of organic chemical reactions is efficiently modelled through the concepts of “electrophiles” and “nucleophiles” (meaning electron-seeking and nucleus-seeking reactive species). On the one hand, an advanced approach of the correlation of the nucleophilicity parameters *N* and electrophilicity *E* has been delivered from the linear free energy relationship log *k* (20 °C) = *s*(*N* + *E*). On the other hand, the general influence of the solvent mixtures, which are very often employed in preparative synthetic chemistry, has been poorly explored theoretically and experimentally, to date. Herein, we combined experimental and theoretical studies of the solvent influence on pyrrolidine nucleophilicity. We determined the nucleophilicity parameters *N* and *s* of pyrrolidine at 20 °C in CH_3_OH/CH_3_CN mixtures containing 0, 20, 40, 60, 80 and 100% CH_3_CN by kinetic investigations of their nucleophilic substitution reactions to a series of 2-methoxy-3-X-5-nitrothiophenes 1a–e (X = NO_2_, CN, COCH_3_, CO_2_CH_3_, CONH_2_). Depending on the resulting solvation medium, the *N* parameters range from 15.72 to 18.32 on the empirical nucleophilicity scale of Mayr. The nucleophilicity parameters *N* first evolve linearly with the content of acetonitrile up to 60% CH_3_CN by volume, but is non linear for higher amounts. We designed a general computation protocol to investigate the solvent effect at the atomistic scale. The nucleophilicity in solvent mixtures was evaluated by combining classical molecular dynamic (MD) simulations of solvated pyrrolidine and a few density functional theory (DFT) calculations of Parr nucleophilicity. The pyrrolidine theoretical nucleophilicity 1/*ω* obtained in various CH_3_OH/CH_3_CN mixtures are in excellent agreement with Mayr's nucleophilicity (*N*) parameters measured. Analyses of the molecular dynamic trajectories reveal that the decrease of the nucleophilicity in methanol rich mixtures arises predominantly from the solvation of the pyrrolidine by methanol molecules through strong hydrogen bonds. Last, we proposed a simple model to predict and accurately reproduce the experimentally obtained nucleophilicity values.

## Abstract 2

Combined experiments and modelling rationalize the large influence of solvent composition on pyrrolidine nucleophilicity.

## Graphical abstract

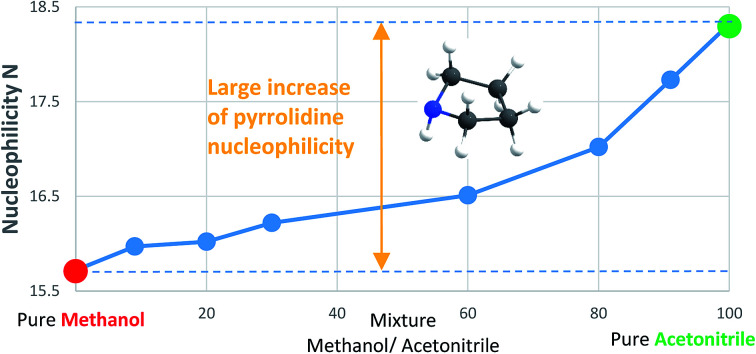

## 1 Introduction

The course of organic chemical reactions is efficiently modelled through the concepts of “electrophiles” and “nucleophiles”.^1^ These important concepts were quickly embraced by the chemical community, and empirical approaches were proposed by many authors both experimentally^2–4^ and theoretically^5,6^ to obtain quantitative scales.

In particular, the nucleophilicity parameters *N* and electrophilicity *E*, as introduced by Mayr group, allows to quantitatively predict the rate constant between an electrophile and a nucleophile based on a linear free energy relationship eqn (1) (ref. 4–10) in which *k* corresponds to the second-order rate constant, *N* and *s* are nucleophile-specific parameters, and *E* is the electrophilicity parameter.

1 log *k* (20 °C) = *s*(*E* + *N*)

While the electrophilicity of a molecule is almost independent of the solvent, the nucleophilicity can strongly influenced by it.^11^ For example, the nucleophilicity parameter *N* of 4-(dimethylamino)pyridine jumps from 13.19 in water to 15.80 in dichloromethane.^12^ Being quantitative, Mayr's approach (eqn (1)) is an attractive entrance to study the general influence of solvent mixtures which are classically employed in preparative synthetic chemistry and was recently used for the synthesis of nanolympiadane.^13^

Previous studies have shown that the dependence of the nucleophilicity parameter *N* on the solvent mixture can be greatly non linear^14^ so that it appears difficult to predict the rate of a nucleophile–electrophile reaction in a new solvent or a new solvent mixture. Therefore, a careful study of solvent influence appeared to us indispensable for providing further advances to the general effort of scaling and predicting nucleophilic and electrophilic parameters.

As a starting point to develop a general approach, we choose to study electrophile/nucleophile reactivity of thiophenes and pyrrolidine into two miscible solvents (acetonitrile, methanol), prepared in mixture at various ratio.

On one hand, pyrrolidine and imidazolidinone derivatives are the object of current interest as organocatalysts used in contemporary organic synthesis.^15^ Their intrinsic reactivity has been examined in relation with bioactive compounds of natural or synthetic origin, which incorporate such nucleophilic scaffolds.^16–19^ Recently, Mayr group investigated the kinetics of the reactions of several pyrrolidine derivatives and they integrated the resulting constitutive parameters of pyrrolidines into the so-called Mayr's nucleophilicity scale.^16^

On the other hand, thiophenes are heterocycles of industrial interest,^20,21^ and substituted thiophenes are scaffold found within pharmaceuticals, conductive polymers, photochromic molecular switches, liquid crystals, *etc*.^20^ Regarding the general studies on nucleophilic and electrophilic parameters of heterocycles, Boubaker group investigated the reactions of 2-methoxy-3-X-5-nitrothiophenes electrophiles 1a–e (where X = NO_2_, CN, COCH_3_, CO_2_CH_3_ and CONH_2_) with a variety of N-based nucleophiles, in different solvents at 20 °C.^22,23^ The derived second-order rate constants have been employed to determine the reactivity parameters of these series of thiophenes 1a–e, according to the Mayr's linear free energy relationship. The electrophilic parameter *E* values of 1a–e have been found ranging from −19.09 to −15.26, going respectively, from 1e (X = CONH_2_) the least reactive thiophene derivative, to 1a (X = NO_2_), the most reactive specimen.

This set of results and collected data has been of high interest for the understanding of both individual intrinsic organic reactivity and mutual interaction of pyrrolidine and thiophene derivatives.^17,22,23^

The present work aimed at determining the nucleophile specific parameters *N* and *s* of pyrrolidine, as a model, in CH_3_OH/CH_3_CN mixtures. The changes in nucleophilicity parameters *N* values as a function of acetonitrile content was used to investigate the solvent-mixture overall effect at the atomistic level by combining experimental and theoretical approaches. As methanol and acetonitrile dielectric constant are rather close, a continuum model is not sufficient to properly describe the solvation effect on the nucleophilicity. To appreciate the effects of solvent on the parameters of nucleophilicity of pyrrolidine, we performed classical molecular dynamic simulations of a pyrrolidine molecule solvated by a mixture of methanol and acetonitrile solvent molecules. Our studies confirmed the strong dependence of the nucleophilicity (and thus reaction rate) on the nature of the solvation medium. In the present case it comes from a gradual methanol desolvation to pyrrolidine involving four to zero molecules when the acetonitrile amount is gradually increased. Our study provides a relevant model for the more systematic inclusion of varied solvent into the reactivity studies of valuable electrophile/nucleophile organic reagents such the present heteroaromatics.

## 2 Experimental section

### 2.1 Materials

The thiophenes 1a–e were prepared as previously described.^24–26^ Pyrrolidine was received from commercial source and distilled before use. Acetonitrile and methanol HPLC grade >99.9% were used without further purification.

### 2.2 Kinetic measurements

The kinetic study was performed using a spectrophotometer (UV-1650 Shimadzu) equipped with a Peltier temperature controller (TCC-240 A), which is able to keep constant temperature within 0.1 K. The reactions were carried out under pseudo-first order conditions in which the pyrrolidine concentration (6 × 10^−4^ to 8 × 10^−1^ mol L^−1^) was at least 20 times greater than the substrate concentration (about 3 × 10^−5^ mol L^−1^). The first-order rate constants measured, *k*_obsd_, values, together with detailed reaction conditions, are summarized in Tables S1–S6 in the ESI.† Reproducible kinetics constants were measured from several consistent experimental runs within ±3–5% standard deviation (Table S7 in the ESI†).

### 2.3 Theoretical models and computational details

All quantum calculations were performed in the framework of density functional theory (DFT) by using the Gaussian 09 software package.^27^ Energies and forces were computed with the B3LYP functional^28,29^ empirically corrected for dispersion effects using the D3 scheme of Grimme with the Becke–Johnson damping.^30^ The bulk effect of the solvent was described using a polarizable continuum model as implemented in Gaussian09. Geometry optimizations without symmetry constraints and the corresponding frequency calculations were conducted with the 6-311+G(d,p) basis set for all atoms.^31^

Methanol and acetonitrile dielectric constant are fairly close with *ε*_r_ = 32.613 for methanol and *ε*_r_ = 35.688 for acetonitrile, thus a continuum model is not sufficient to properly describing the solation effect on the nucleophilicity. To more precisely appreciate the effects of solvent on the parameters of nucleophilicity of pyrrolidine, we performed molecular dynamic simulations with the Amber simulation suite of a pyrrolidine molecule explicitly solvated by a mixture of methanol and acetonitrile solvent molecules.^32^ Pure methanol, pure acetonitrile and mixture containing 9%, 20%, 40%, 50%, 60%, 80% and 90% of acetonitrile in methanol were simulated. The force field parameters and the technical details of the simulation are given in ESI Fig. S2 and S3.† For each simulation, we computed the distribution methanol molecules in the first solvation sphere or pyrrolidine. All calculation results were grouped in Tables S11 and S12 in the ESI.† These simulations showed that the first layer of solvation contains between zero and four methanol molecules.

We computed the Parr nucleophilic indices *ω*^−1^ of pyrrolidine surrounded by *n* = 0, 1, 2, 3 and 4 explicit methanol molecules while the bulk solvation effects were described by a continuum. According to Parr,^5^ the nucleophilicity index is the inverse of the electrophilicity index *ω* that can be estimated using:2
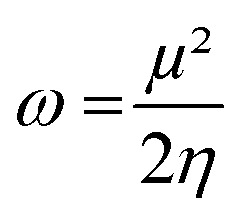


In which *μ* is the chemical potential and *η* the global hardness^6^ of pyrrolidine. Both can be evaluated in the context of DFT:3
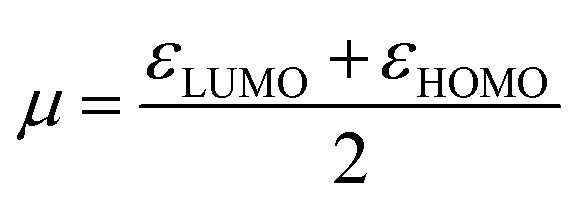

4 *η* = *ε*_LUMO_ − *ε*_HOMO_

The pyrrolidine nucleophilicity are given in Table 1. These values are averaged using the distribution of methanol molecules in the first solvation layer of pyrrolidine obtained in the MD simulations (see ESI Table S10, Fig. S4 and S5†).

**Table 1:** Nucleophilic parameters *ω*^−1^ of pyrrolidine surrounded by *n* explicit methanol molecules (in a acetonitrile PCM) computed at the B3LYP/6-311 ++ G (d, p) level

Number of explicit methanol	0	1	2	3	4	5
Nucleophilic indices (*ω*^−1^)	30.14	28.66	27.85	27.68	27.77	27.28

## 3 Results and discussion

### 3.1 Kinetics of the reactions of thiophenes 1a–e with pyrrolidine

**Scheme 1:**
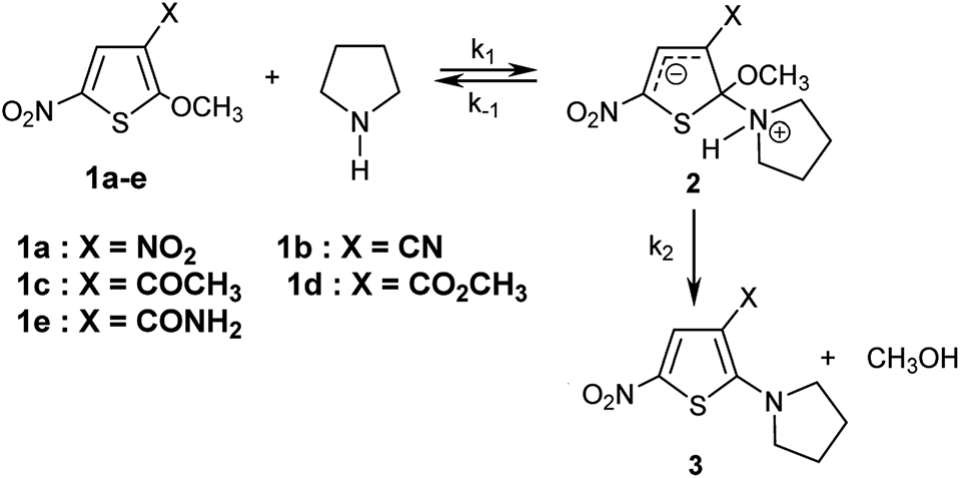
Reactions of 2-methoxy-3-X-5-nitrothiophenes 1a–e with pyrrolidine in CH_3_OH/CH_3_CN- mixtures at 20 °C.

The kinetics of the reactions of the series of thiophenes 1a–e employed as reference electrophiles with pyrrolidine (Scheme 1) are collected in Table 2.^22,23^

**Table 2:** Electrophilicity parameters *E* and p*K*_a_ for the thiophenes 1a–e used as reference in this work

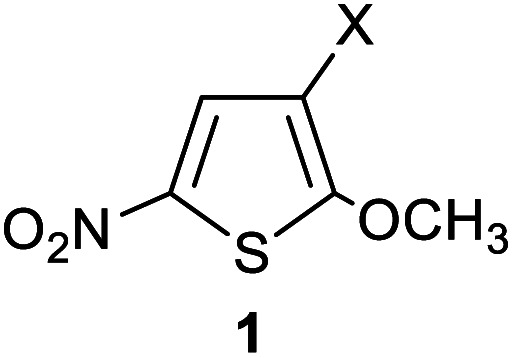	*E*	p*K*_a_^a^
1a: X = NO_2_	−15.26^a^	11.36
1b: X = CN	−16.60^a^	12.02
1c: X = COCH_3_	−17.65^a^	12.82
1d: X = CO_2_CH_3_	−18.48^b^	13.34
1e: X = CONH_2_	−19.09^b^	13.46

a From ref. 22.

b From ref. 23.

**Fig. 1:**
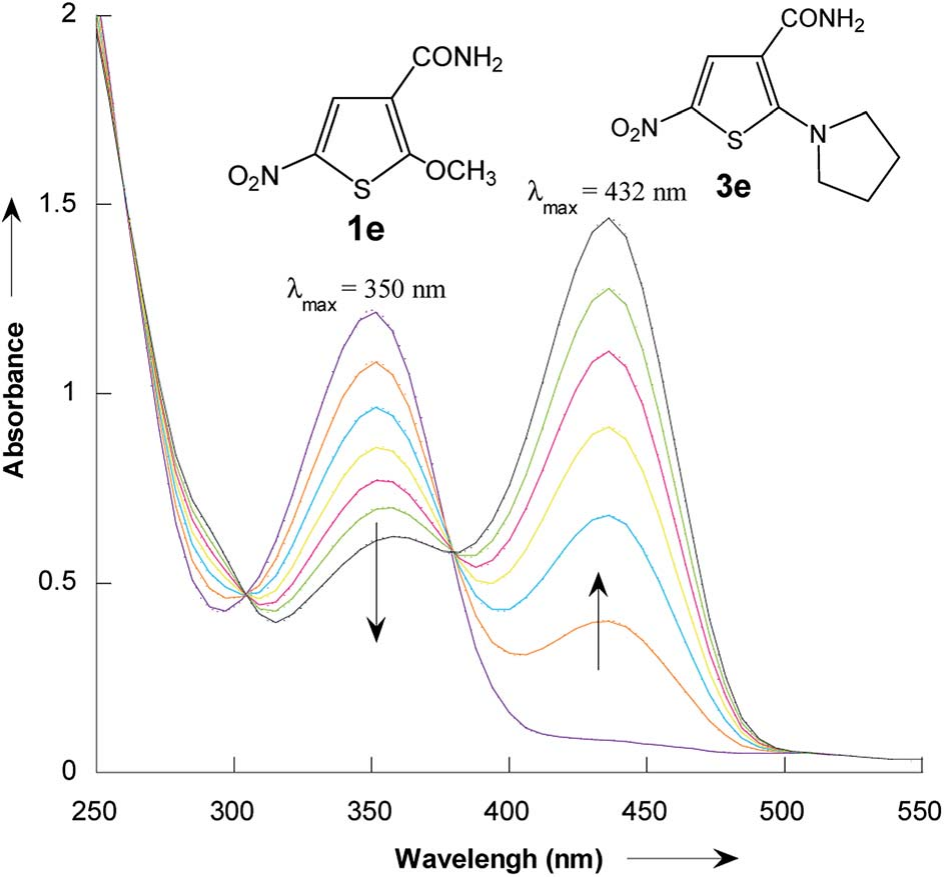
Time dependence of the electronic absorption spectrum of thiophene 1e (3 × 10^−5^ mol L^−1^) in the presence of pyrrolidine (10^−1^ mol L^−1^) in methanol at 20 °C.

The reactions of 2-methoxy-3-X-5-nitrothiophenes 1a–e with pyrrolidine were followed spectrophotometrically by monitoring the formation of the products 3a–e at their absorption maxima (432–540 nm). An illustrative example is given in Fig. 1, which shows the set of UV-visible absorption spectra describing the progressive conversion of 1e to the product 3e (X = CONH_2_) resulting of the nucleophilic addition of pyrrolidine.

**Fig. 2:**
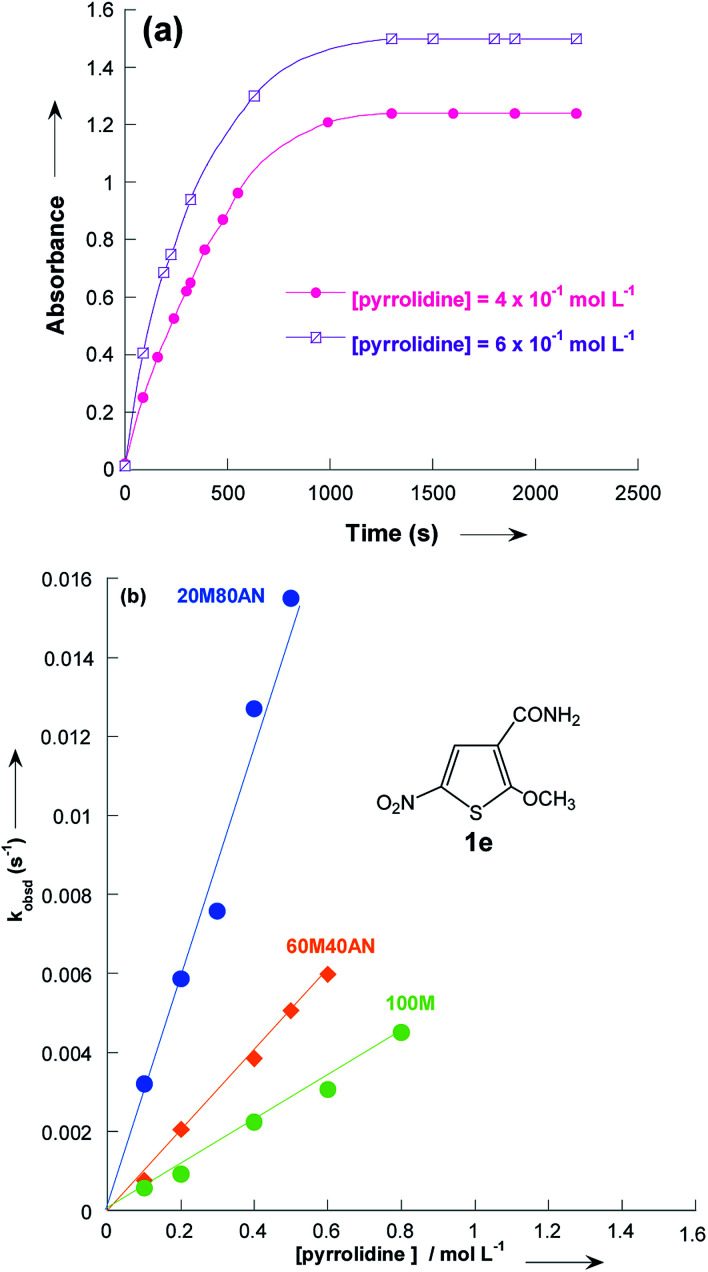
(a) Plot of the absorbance at *λ*_max_ = 432 nm *vs.* time of thiophene 1e with pyrrolidine in methanol at 20 °C. (b) Influence of pyrrolidine concentration on the observed pseudo first-order rate constants *k*_obsd_ for addition to thiophene 1e in methanolic CH_3_OH/CH_3_CN solutions at 20 °C. Mixtures of solvents given as (v/v). Abbreviations used for the solvents are AN (CH_3_CN) and M (CH_3_OH).

In all experiments, only one relaxation time was observed when the substitution products 3a–e were generated in the presence of a large excess of pyrrolidine. Typical results are given in Fig. 2a. The observed first-order rate constants k_obsd_ were obtained from the correlation ln(*A*_∞_ − *A*_*t*_) against time, where *A*_∞_ and *A*_*t*_ are the values of absorbance at the equilibrium and at time *t*, respectively. The plots of *k*_obsd_*versus* pyrrolidine concentrations are linear with *R*^2^ > 0.9987 passing through the origin. This indicates that the reactions are not base catalysed, and that the formation of the intermediate zwitterion 2 is rate determining (Scheme 1).^22,23,33–35^ The second-order rate constants *k*_1_ (mol^−1^ L s^−1^) for all reactions, which are listed in Table 3, can be readily derived from eqn (5).

5 *k*_obsd_ = *k*_1_ [pyrrolidine]

**Table 3:** Effect of substituent and solvent on the second-order rate constants *k*_1_ (L mol^−1^ s^−1^) for the S_N_Ar reactions of 2-methoxy-3-X-5-nitrothiophenes 1a–e with pyrrolidine in CH_3_OH/CH_3_CN mixtures at 20 °C

% CH_3_CN by volume	0	20	40	60	80	100
1a: X = NO_2_	1.20	2.33	3.01	4.96	10.8	—
1b: X = CN	1.66 × 10^−1^	2.85 × 10^−1^	4.74 × 10^−1^	7.41 × 10^−1^	1.48	10.9
1c: X = COCH_3_	3.89 × 10^−2^	5.72 × 10^−2^	1.04 × 10^−1^	1.51 × 10^−1^	2.88 × 10^−1^	1.91
1d: X = CO_2_CH_3_	1.15 × 10^−2^	2.08 × 10^−2^	3.74 × 10^−2^	5.15 × 10^−2^	9.53 × 10^−2^	8.14 × 10^−1^
1e: X = CONH_2_	5.60 × 10^−3^	7.00 × 10^−3^	1.03 × 10^−2^	1.72 × 10^−2^	3.09 × 10^−2^	3.32 × 10^−1^

### 3.2 Effect of the solvent mixture on the nucleophilic reactivity of the pyrrolidine

**Fig. 3:**
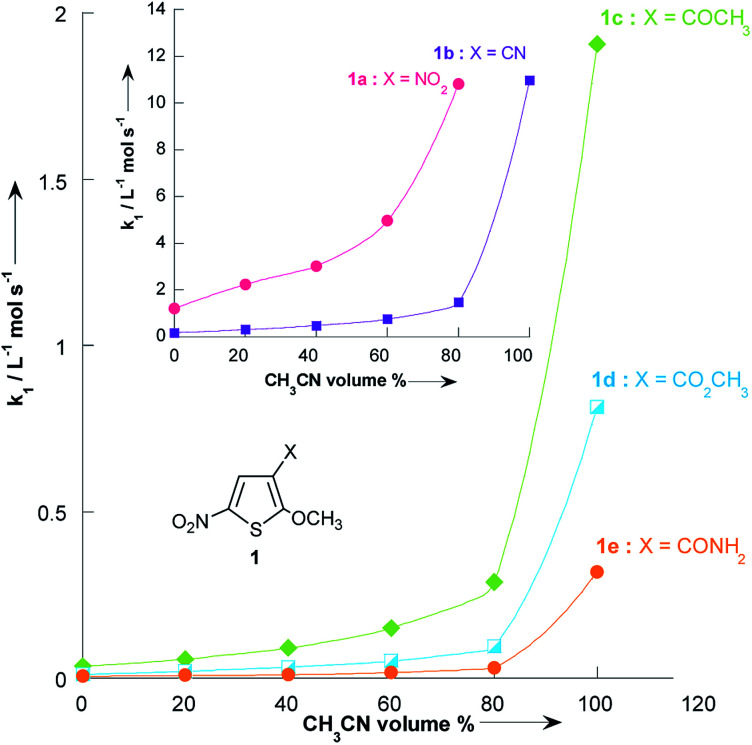
Plots of the second-order rate constants for the reactions of pyrrolidine with the 2-methoxy-3-X-5-nitrothiophenes 1a–e against volume % of CH_3_CN at 20 °C. Data from Table 3.

Fig. 3 and Table 3 show the contrasting changes in the reactivity of thiophenes 1a–e on transfer from CH_3_OH to CH_3_CN as a function of the amount of CH_3_CN in volume%. Similar curves are also obtained as a function of the CH_3_CN molar fractions, as shown in Fig. S1 and Table S9.† As can be seen, the second-order rate constants, *k*_1_, depend nonlinearly on the CH_3_CN volume%. The rate constant *k*_1_ significantly increases in the CH_3_CN-rich section and changes very little in the 0–60% CH_3_CN section. Surprisingly, *k*_1_ decreases while the polarity of the solvent mixture increase: the observed reactivity sequence being *k*_1_ [CH_3_OH] < *k*_1_ [80% CH_3_OH/20% CH_3_CN] < *k*_1_ [60% CH_3_OH/40% CH_3_CN] < *k*_1_ [40% CH_3_OH/60% CH_3_CN] < *k*_1_ [20% CH_3_OH/80% CH_3_CN] < *k*_1_ [CH_3_CN].

**Scheme 2:**
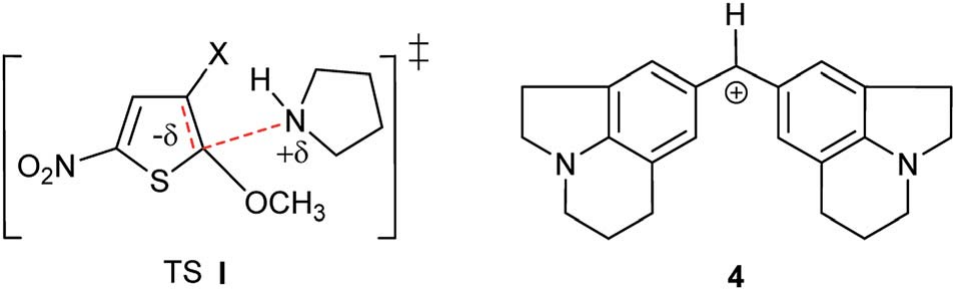
Transition Structure I and cation 4.

This behaviour might first seem in contradiction with the fact that this reaction is known to proceed *via* a dipolar transition state (TS I, Scheme 2), which should be more stabilized in methanolic solutions.^36,37^ However, as will be further shown later, it results from the progressive desolvation of pyrrolidine with decreasing CH_3_OH content upon addition of CH_3_CN.^38,39^ A comparable effect has been reported by Mayr and co-workers in the reactions of benzhydrylium cation (Scheme 2) with OH^−^ in H_2_O–CH_3_CN mixtures.^40^

We further examined the solvation medium effect on the nucleophilic reactivity in terms of changes in solvent nucleophilicity parameters N_1_ using CH_3_OH/CH_3_CN mixtures.

We employed the linear dependence of the solvent nucleophilicity N_1_ on the amount of % CH_3_CN volume to interpolate the values of N_1_ for 40% and 60% of acetonitrile in methanol from the values reported in literature^41^ (see ESI†). N_1_ values are collected in Table 4.

**Table 4:** Solvent nucleophilicity parameter *N*_1_ values for various CH_3_OH/CH_3_CN mixtures at 20 °C

% CH_3_CN by volume	0	20	40	60	80
Solvent nucleophilicity parameter N_1_	7.54^a^	7.20^a^	6.84^b^	6.47^b^	6.04^a^

a From ref 41.

b Values interpolated from ref 41, see Table S8 in ESI.

**Fig. 4:**
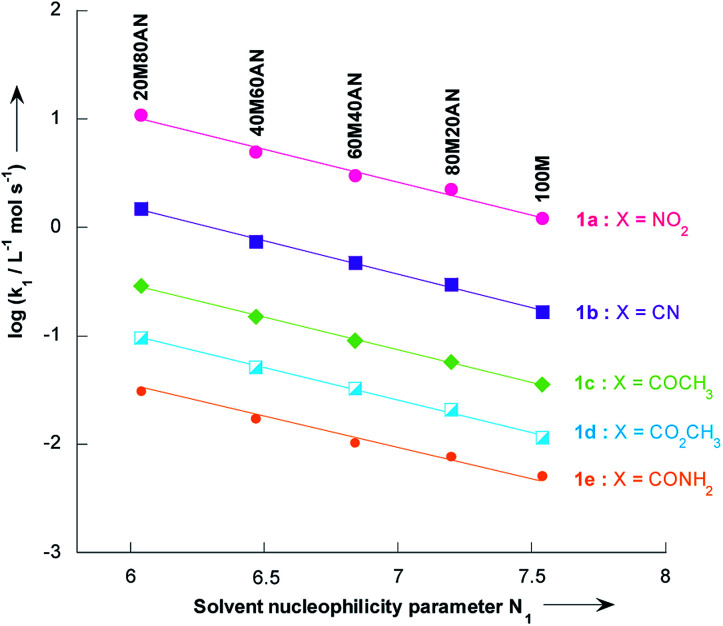
Plots of the second-order rate constants for the reactions of pyrrolidine with the 2-methoxy-3-X-5-nitrothiophenes 1a–e against solvent nucleophilicity parameter *N*_1_ at 20 °C. Mixtures of solvents given as (v/v). Abbreviations used for the solvents are AN (CH_3_CN) and M (CH_3_OH). Data from Tables 3 and 4

In contrast to the nonlinear relationships observed between log *k*_1_ and %CH_3_CN volume, excellent correlation coefficients (*R*^2^ > 0.9931) were found in all systems when the log *k*_1_ values were plotted *versus* the solvent nucleophilicity parameter N_1_,^41^ for solutions having *N*_1_ > 6.04 (*i.e.* less than 80% vol. CH_3_CN, Fig. 4). This result implies that the effect of solvent nucleophilicity is practically independent of the electronic nature of the substituent X. Most importantly, these linear correlations can be used to predict the unknown second-order rate constants, *k*_1_, values for reactions of thiophenes 1a–e with pyrrolidine in a given CH_3_OH/CH_3_CN mixture. This is particularly the case in CH_3_OH/CH_3_CN mixtures containing 9, 33, 50 and 67% CH_3_CN where the values of *k*_1_ for all thiophenes 1a–e have thus been obtained by extrapolation of the corresponding N_1_ data reported by Minegishi and co-worker^41^ (see Table S8 in the ESI†).

### 3.3 Mayr's nucleophilicity (*N*) parameters of pyrrolidine in methanolic acetonitrile solutions

The electrophilicity parameters *E* for the five thiophenes 1a–e,^22,23^ were employed to quantify the nucleophilicity parameters *N* and *s* of pyrrolidine in methanol/acetonitrile solutions.

**Fig. 5:**
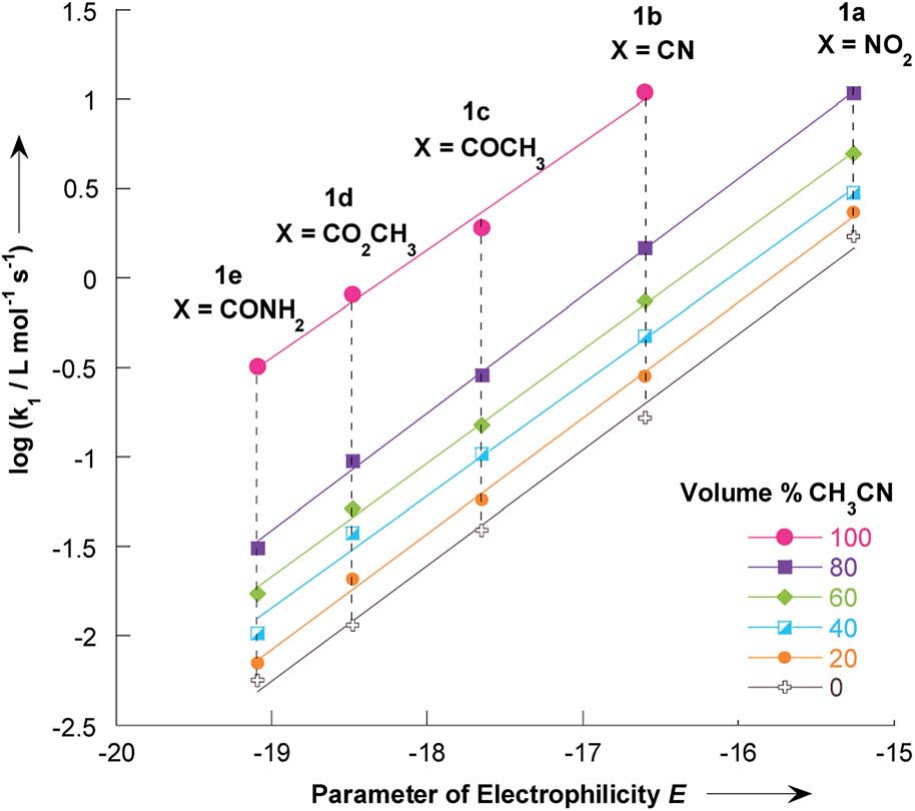
Correlations of the second-order rate constants (20 °C) for the reactions of pyrrolidine with the 2-methoxy-3-X-5-nitrothiophenes 1a–e in various CH_3_OH/CH_3_CN mixtures toward the electrophilicity parameters *E*. Data from Tables 1 and 2.

Using the data given in Tables 1 and 2, plots of log *k*_1_*versus* electrophilicity parameters *E* of 1a–e have been constructed. As can be seen in Fig. 5, linear correlations were obtained, which yield the nucleophilicity parameters *N* and *s*, as defined by the linear free energy relationship (1). The nucleophilicity parameters *N* and *s* for pyrrolidine in various methanol/acetonitrile mixtures at 20 °C are reported in Table 5.

**Table 5:** Nucleophilicity (*N*) and slope (*s*) parameters for pyrrolidine in various CH_3_OH/CH_3_CN mixtures (v/v) at 20 °C

% CH_3_CN	0	9	20	40	60	80	100
*N*	15.72	15.85^a^ (15.97)^b^	16.02	16.22	16.51	17.02	18.32 (18.64)^c^
*s*	0.64	0.64^b^	0.64	0.62	0.63	0.65	0.61 (0.60)^c^

a Value calculated according eqn (6).

b From ref. 44.

c From ref. 42.

The *N* value of 18.32 experimentally found in pure acetonitrile is consistent with the value of 18.64 reported by Kanzian and co-workers in the same solvent.^42^ It appears that adding acetonitrile to a methanolic solution of pyrrolidine resulted in only small changes in nucleophile specific parameter *s*.

The transfer from CH_3_CN to CH_3_OH corresponds to a relatively important decrease in the nucleophilicity of pyrrolidine (Table 5, Δ*N* = 2.60). This decrease of the nucleophilicity values in the CH_3_OH rich solvation medium could be attributed to a stronger solvation of pyrrolidine in this protic solvent, as confirmed below.

**Fig. 6:**
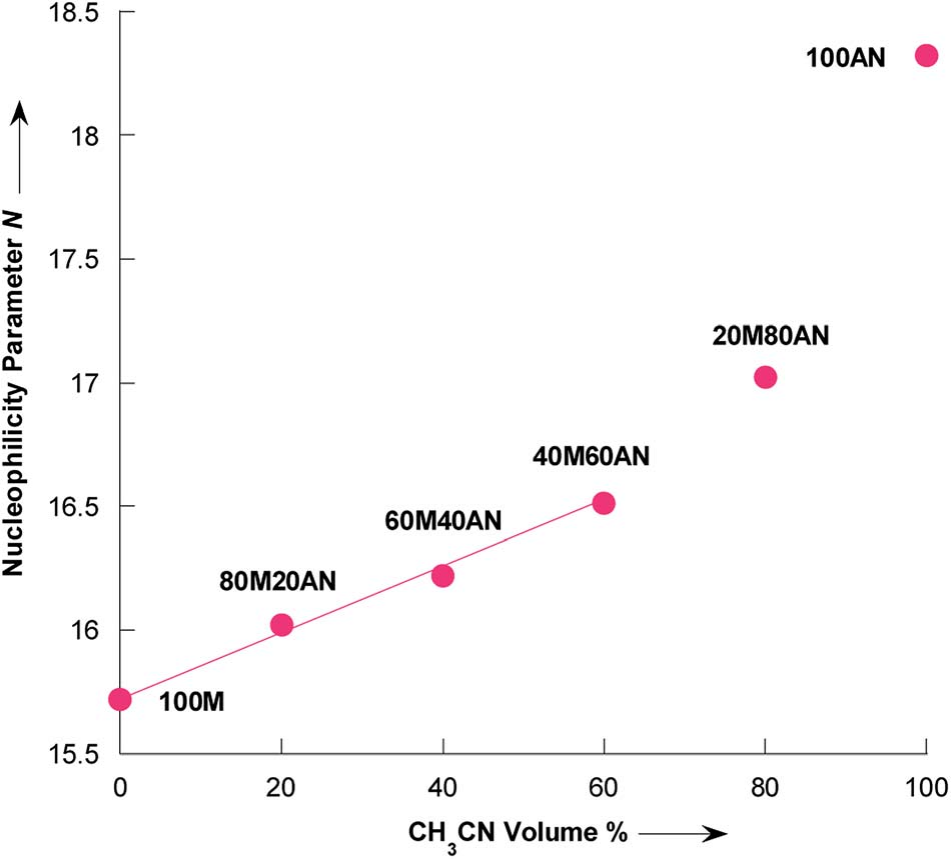
Dependence of the nucleophilicity parameter N of pyrrolidine on the composition of CH_3_OH/CH_3_CN mixtures at 20 °C. Dada from Table 4. Mixtures of solvents are given as (v/v), solvents: M = CH_3_OH and AN = CH_3_CN.

Literature data indicated a nucleophilicity parameter for piperidine of 17.35 in CH_3_CN,^42^ whereas the piperidine appears more nucleophilic in pure water (*N* = 18.13).^43^ For morpholine the *N* value is essentially the same in pure CH_3_CN and H_2_O: *N*_(H_2_O)_ = 15.62,^43^*N*_(CH_3_CN)_ = 15.65.^42^ The changes in nucleophilicity parameter of pyrrolidine observed herein were also examined as a function of the variation in volume fraction of acetonitrile (%CH_3_CN). As seen from the Fig. 6, *N* linearly correlates with the vol% of CH_3_CN up to approximately 60% vol. CH_3_CN, following the eqn (6) with *R*^2^ = 0.9972:

6 *N* = 15.732 + 0.0129% CH_3_CN

Eqn (6) validity is supported by comparing with the experimental *N* value in the mixture of 91% CH_3_OH/9% CH_3_CN as reported by Phan *et al.*,^44^ to the interpolated value according eqn (6), as shown in Table 5. There is an excellent agreement between experimentally determined and calculated values with the average absolute error being only 0.12 *N* units.

### 3.4 Theoretical nucleophilicity (1/*ω*) parameters of pyrrolidine in methanolic mixtures with acetonitrile

**Fig. 7:**
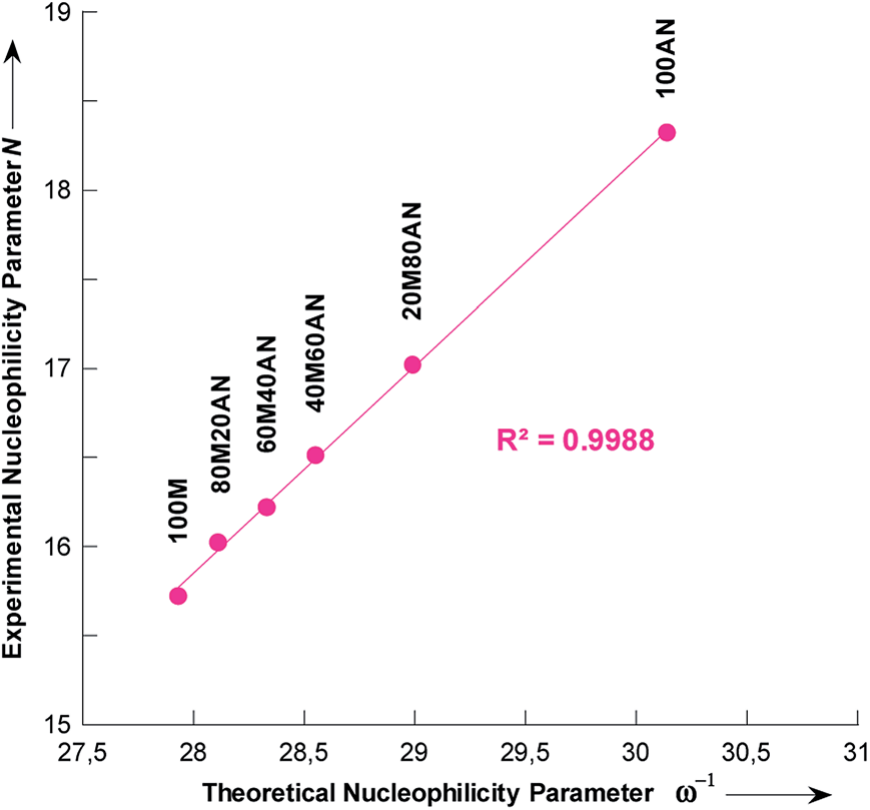
Plot of the Mayr nucleophilicity (*N*) parameters of pyrrolidine *vs.* theoretical nucleophilicity (1/*ω*) calculated at B3LYP/6-311G(d,p) coupled with molecular dynamic simulation on the composition of CH_3_OH/CH_3_CN mixtures. Mixtures of solvents are given as (v/v), solvents: M = CH_3_OH and AN = CH_3_CN. *R*^2^ is the regression coefficient.

In the simulation process the number of methanol molecules *n* varies, and we found that on average one pyrrolidine molecule is surrounded by two molecules in pure methanol. This number decreases to zero when the content of the acetonitrile mixture is increased (Fig. S5†). In Table 6 is collected the theoretical nucleophilicity 1/*ω* of pyrrolidine in various methanolic CH_3_CN solutions, together with the Mayr's nucleophilicity (*N*) parameters measured in this study. A very good correlation was found, *R*^2^ = 0.9988, between theoretical 1/*ω* and experimental *N* values (Fig. 7),^45–47^ which is defined by eqn (7).

7 *N* = 1.1629 × *ω*^−1^ − 16.712

**Table 6:** Comparison between the experimental (*ω*^−1^ and *N* obtained from eqn (7)) and theoretical nucleophilicity parameters of pyrrolidine in various CH_3_OH/CH_3_CN mixtures (v/v)

% CH_3_CN	0	9	20	40	60	80	91	100
*Ω* ^−1^	27.93	28.01	28.11	28.33	28.55	28.99	29.37	30.14
*N* from eqn (7)	15.77	15.86	15.98	16.23	16.49	17.00	17.44	18.34
*N*	15.72	15.85^a^ (15.97^b^)	16.02	16.22	16.51	17.02	17.73^b^	18.32
*N*_mod_	15.88	15.94	16.03	16.24	16.57	17.13	17.65	18.32

a From eqn (6).

b Experimental nucleophilicity values were taken from ref. 44.

Chamorro and co-workers have observed a linear correlations between the Mayr's nucleophilicity (*N*) parameters for a series of primary and secondary amines and the theoretical nucleophilicity index (*ω*^−^) obtained at the DFT level.^45^

The validity of eqn (7) was checked by estimating the nucleophilicity parameters *N* of pyrrolidine in CH_3_OH/CH_3_CN mixtures containing 9 and 91% CH_3_CN that had been experimentally measured.^44^ The detailed results are listed in Table 6. These results clearly show that the predicted values of *N* are in excellent agreement with the experimental data.

### 3.5 Modelling influence of the solvation

**Fig. 8:**
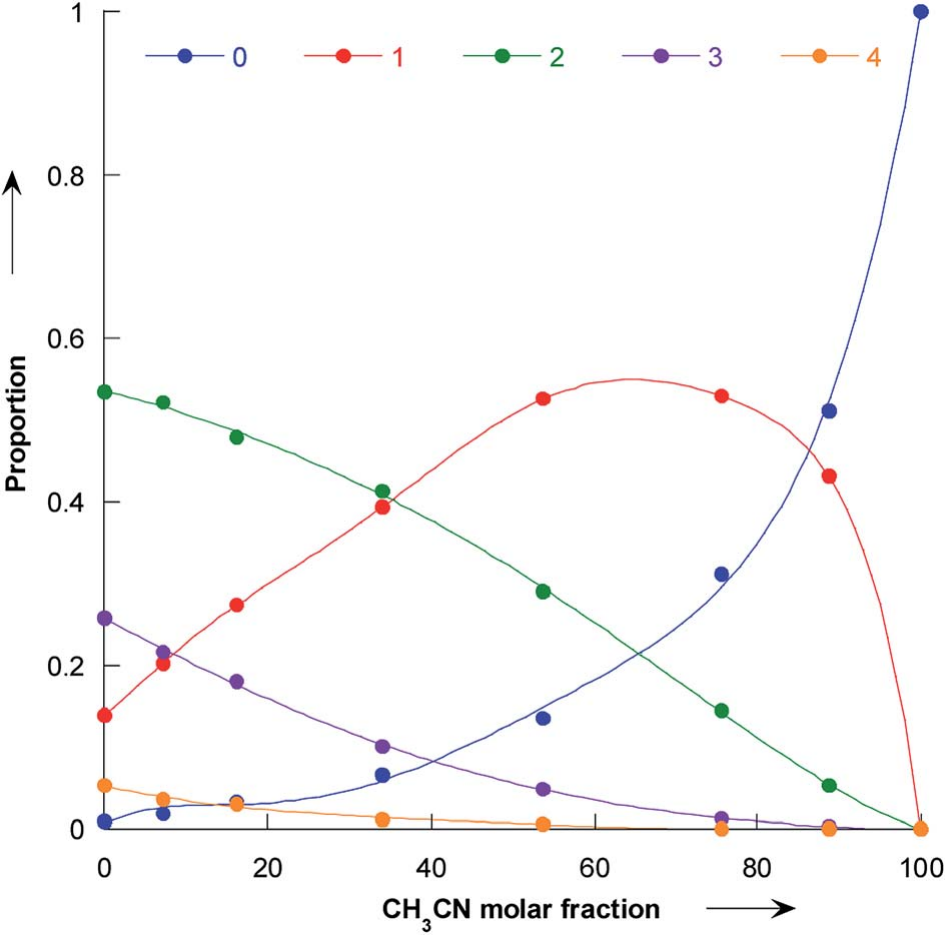
Plot of the proportion of bare pyrrolidine (dark blue), and pyrrolidine solvated by 1 (red), 2 (green), 3 (purple) and 4 (orange) molecules as a function of the molar fraction of acetonitrile.

Our simulations reveal that the evolution of the pyrrolidine nucleophilicity on the composition of the methanol/acetonitrile mixture mainly comes from the fact that methanol forms a strong hydrogen bonds with the nitrogen lone pair and the NH bond of the pyrrolidine. Solvated molecules are much less nucleophilic: bare pyrrolidine nucleophilicity is 18.32 while it is only 15.67 for the bis-methanol pyrrolidine and 15.01 for the pyrrolidine solvated by five methanol. The proportion of pyrrolidine solvated by 0, 1, 2, 3 and 4 methanol molecules is shown in Fig. 8 as a function of the acetonitrile molar fraction.

As the amount of methanol in the mixture is decreased, the amount of bare pyrrolidine molecule increases and so does the observed nucleophilicity. In particular, for molar fraction greater than 60%, the bare pyrrolidine is more abundant than the bis-methanol pyrrolidine. As a consequence, the average pyrrolidine nucleophilicity parameter *N* rises steeply, following the amount of unsolvated pyrrolidine.

It is interesting to note that solvated molecules have similar nucleophilicities, so that we could model the solvation process as a two states system:

8 Pyr + M ⇌ (M–Pyr)

The equilibrium constant of eqn (8) is denoted by 
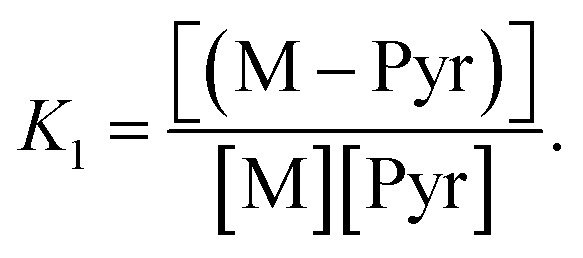
 We decided to set the nucleophilicity of Pyr and (M–Pyr) to 18.32 and 15.01 respectively, and adjusted *K*_1_ on the experimental nucleophilicities: Ke_1_ = 0.11355.

Using the definition of *K*_1_, the observed nucleophilicity is finally:9
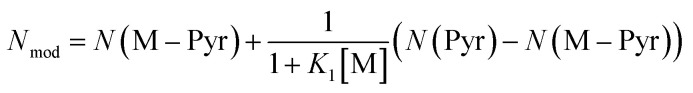


Results from *N*_mod_ are reported in Table 6. The very good agreement with the experimental values confirmed that the influence of the solvent on nucleophilicity can be modelled by our two states approach.

## 4 Conclusions

The reactions of 2-methoxy-3-X-5-nitrothiophenes 1a–e with pyrrolidine were studied kinetically by UV-visible spectroscopy in CH_3_OH, CH_3_CN and various CH_3_OH/CH_3_CN vol/vol. mixtures at 20 °C. The nucleophilicity parameters *N* and *s* for pyrrolidine in CH_3_OH/CH_3_CN mixtures of different compositions as defined by Mayr Equation log *k* (20 °C) = *s*(*E* + *N*) have been determined and found to cover a range from 15.72 to 18.32. We have shown that the nucleophilicity parameters *N* for pyrrolidine are linearly related to the amount of acetonitrile (in % CH_3_CN volume) for ratio less than 60%. Finally, with the *N* and *s* values determined, it becomes possible to make predictions of second-order rate constants for reactions of pyrrolidine with others electrophiles of known *E* parameters. The nucleophilicity index 1/*ω* for pyrrolidine in solvent mixture of acetonitrile and methanol have been determined by combining DFT resampling of classical MD simulations. The theoretical values agree with the experimental ones, and the experimental dependence of the nucleophilicity parameters of pyrrolidine on mixture solvent methanol/acetonitrile was confirmed and supported by the simulation. The net dependence of the nucleophilicity parameter of pyrrolidine on solvent is mainly explained by a gradual methanol desolvation when the amount of acetonitrile is increased. The correlation between the experimental and theoretical values obtained herein indicates that this theoretical approach could be further processed to predict the nucleophilicity parameters of other nucleophiles in solvent mixtures and ultimately an automated tool for tuning electrophilic–nucleophilic reactivity as a function of a precise mixture of binary solvent. Further extension might concern ternary mixture of solvents.

## Conflicts of interest

There are no conflicts to declare.

## Supplementary Material

RA-010-D0RA06324J-s001
