# Supplementary material for: Influence of solvent mixture on nucleophilicity parameters: the case of pyrrolidine in methanol–acetonitrile
Source: RSC Adv. 2020 Aug 3;10(48):28635–43. doi: 10.1039/d0ra06324j (PMC9055836; doi:10.1039/d0ra06324j)
Supplement: RA-010-D0RA06324J-s001 [file RA-010-D0RA06324J-s001.pdf]

Electronic Supplementary Material (ESI) for RSC Advances.
This journal is © The Royal Society of Chemistry 2020

Supporting Information
For
Influence of Solvent Mixture on Nucleophilicity Parameters: The Case of Pyrrolidine in Methanol-Acetonitrile

Salma SOUISSI, Wahiba GABSI, Abderraouf ECHAIEB, Julien ROGER, Jean-Cyrille HIERSO, Paul FLEURAT-LESSARD,* and Taoufik BOUBAKER*

Table of contents

Experimental rate constants 2
Table S 1: The k_{obds} and k_1 values for the reactions of 2-methoxy-3-X-5-nitrothiophene 1a-e with pyrrolidine in pure methanol at 20 °C. 2
Table S 2: The k_{obds} and k_1 values for the reactions of 2-methoxy-3-X-5-nitrothiophene 1a-e with pyrrolidine in 80 % methanol - 20 % acetonitrile at 20 °C. 3
Table S3: The k_{obds} and k_1 values for the reactions of 2-methoxy-3-X-5-nitrothiophene 1a-e with pyrrolidine in 60 % methanol - 40 % acetonitrile at 20 °C. 4
Table S 4: The k_{obds} and k_1 values for the reactions of 2-methoxy-3-X-5-nitrothiophene 1a-e with pyrrolidine in 40 % methanol - 60 % acetonitrile at 20 °C. 5
Table S 5: The k_{obds} and k_1 values for the reactions of 2-methoxy-3-X-5-nitrothiophene 1a-e with pyrrolidine in 20 % methanol - 80 % acetonitrile at 20 °C. 6
Table S 6: The k_{obds} and k_1 values for the reactions of 2-methoxy-3-X-5-nitrothiophene 1b-e with pyrrolidine in pure acetonitrile at 20 °C 7
Table S 7: Values of second-order rate constants extrapolated from data of this work (see Figure 4) for the S_NAr reactions of 2-methoxy-3-X-5-nitrothiophenes 1a-e with pyrrolidine in CH_3CN/CH_3OH mixtures containing 9, 33, 50 and 67% CH_3CN at 20 °C. 8
Interpolating solvent Nucleophilicity N_1 8
Table S 8: Solvent nucleophilicity N1 for different methanol/acetonitile ratios, from Ref 1. 8
Using acetonitrile molar fractions 8
Figure S 1: Plots of the second-order rate constants for the reactions of pyrrolidine with the 2-methoxy-3-X-5-nitrothiophenes 1a-e against molar fraction of CH_3CN at 20 °C. Data from Table S 9 9
Table S 9: Nucleophilicity parameter N, s for pyrrolidine and molar fraction of acetonitrile as a function of acetonitrile volume% 9
Computational results 10
Computation details of Molecular Dynamic simulations. 10
Table S 10: Number of solvent molecules used to simulate experimental solvent mixtures. 10
Figure S 2: Simulation box for 60% acetonitrile and 40% methanol in volume. The pyrrolidine and methanol in its first solvation layer are shown with ball&stick style. Acetonitrle molecules are shown in blue lines while methanol molecules are shown with red lines. 10
Figure S 3: Radial distribution function $g_{ON}(r)$ for nitrogen atom of the pyrrolidine and the oxygen atom of methanol performed during the last 5ns of MD simulation in pure methanol. 11
Table S 11: Population and average number of methanol molecules (<n>) around pyrrolidine in various mixture solvent methanol-acetonitrile determined at 4 Å radius solvation cage. 12
Table S 12: Probability P(n) of finding n methanol molecule in the first solvation layer of pyrrolidine for different methanol-acetonitrile composition. 12
Figure S 4: Probability of presence of the methanol oxygen around the pyrrolidine. 13
Figure S 5: Average methanol molecules in the first solvation shell of pyrrolidine as a function of the molar fraction of acetonitrile. 13
Table S 13: Probability P(n) of finding n methanol molecule in the first solvation layer of pyrrolidine for different concentration in pyrrolidine. 14
Table S 14: Nucleophilic parameters ω^{-1} of pyrrolidine surrounded by a number n of methanol molecule measured by B3LYP / 6-311 ++ G (d, p) calculation 14
References 15

Experimental rate constants

Table S 1: The k_{obds} and k_1 values for the reactions of 2-methoxy-3-X-5-nitrothiophene **1a-e** with pyrrolidine in pure methanol at 20 °C.

Thiophene	[Pyrrolidine] mol L^{-1}	k_{obds} s^{-1}	k_1 mol^{-1} L s^{-1}	Correlation coefficient
**1a**	2×10^{-3}<br>1×10^{-3}<br>8×10^{-4}<br>7×10^{-4}<br>6×10^{-4}	2.28×10^{-3}<br>1.08×10^{-3}<br>8.63×10^{-4}<br>7.17×10^{-4}<br>5.96×10^{-4}	1.20	0.9998
**1b**	8×10^{-3}<br>6×10^{-3}<br>4×10^{-3}<br>2×10^{-3}<br>1×10^{-3}	1.15×10^{-3}<br>9.18×10^{-4}<br>7.13×10^{-4}<br>3.17×10^{-4}<br>1.54×10^{-4}	1.66×10^{-1}	0.9947
**1c**	8×10^{-2}<br>6×10^{-2}<br>4×10^{-2}<br>2×10^{-2}<br>1×10^{-2}	1.25×10^{-3}<br>2.25×10^{-3}<br>4.62×10^{-3}<br>5.92×10^{-4}<br>6.85×10^{-4}	3.89×10^{-2}	0.9915
**1d**	8×10^{-2}<br>6×10^{-2}<br>4×10^{-2}<br>2×10^{-2}<br>1×10^{-2}	9.50×10^{-4}<br>6.30×10^{-4}<br>4.42×10^{-4}<br>2.46×10^{-4}<br>1.05×10^{-4}	1.15×10^{-2}	0.9945
**1e**	8×10^{-1}<br>6×10^{-1}<br>4×10^{-1}<br>2×10^{-1}<br>1×10^{-1}	4.51×10^{-3}<br>3.06×10^{-3}<br>2.24×10^{-3}<br>9.20×10^{-4}<br>5.70×10^{-4}	5.60×10^{-3}	0.9957

Table S 2: The k_{obds} and k_1 values for the reactions of 2-methoxy-3-X-5-nitrothiophene **1a-e** with pyrrolidine in 80 % methanol - 20 % acetonitrile at 20 °C.

Thiophene	[Pyrrolidine] mol L^{-1}	k_{obds} s^{-1}	k_1 mol^{-1} L s^{-1}	Correlation coefficient
**1a**	6×10^{-4}<br>7×10^{-4}<br>8×10^{-4}<br>1×10^{-3}<br>2×10^{-3}	1.30×10^{-3}<br>1.60×10^{-3}<br>1.80×10^{-3}<br>2.26×10^{-3}<br>4.60×10^{-3}	2.33	0.9998
**1b**	4×10^{-3}<br>6×10^{-3}<br>8×10^{-3}<br>2×10^{-2}<br>4×10^{-2}	1.34×10^{-3}<br>1.91×10^{-3}<br>3.28×10^{-3}<br>5.65×10^{-3}<br>1.19×10^{-2}	2.85×10^{-1}	0.9956
**1c**	8×10^{-3}<br>1×10^{-2}<br>3×10^{-2}<br>5×10^{-2}<br>8×10^{-2}	4.47×10^{-4}<br>5.52×10^{-4}<br>1.81×10^{-3}<br>2.66×10^{-3}<br>4.63×10^{-3}	5.72×10^{-2}	0.9978
**1d**	6×10^{-2}<br>8×10^{-2}<br>1×10^{-1}<br>2×10^{-1}<br>4×10^{-1}	1.30×10^{-3}<br>1.60×10^{-3}<br>2.00×10^{-3}<br>4.30×10^{-3}<br>8.25×10^{-3}	2.08×10^{-2}	0.9995
**1e**	1×10^{-1}<br>2×10^{-1}<br>4×10^{-1}<br>6×10^{-1}<br>8×10^{-1}	5.97×10^{-4}<br>1.39×10^{-3}<br>2.81×10^{-3}<br>4.21×10^{-3}<br>5.60×10^{-3}	7.00×10^{-3}	0.9998

Table S3: The k_{obds} and k_1 values for the reactions of 2-methoxy-3-X-5-nitrothiophene **1a-e** with pyrrolidine in 60 % methanol - 40 % acetonitrile at 20 °C.

Thiophene	[Pyrrolidine] mol L^{-1}	k_{obds} s^{-1}	k_1 mol^{-1} L s^{-1}	Correlation coefficient
**1a**	6×10^{-4}<br>8×10^{-4}<br>1×10^{-3}<br>2×10^{-3}<br>4×10^{-3}	2.28×10^{-3}<br>2.66×10^{-3}<br>3.72×10^{-3}<br>6.44×10^{-3}<br>1.25×10^{-2}	3.01	0.9992
**1b**	1×10^{-3}<br>2×10^{-3}<br>4×10^{-3}<br>2×10^{-2}<br>4×10^{-2}	4.74×10^{-4}<br>9.48×10^{-4}<br>1.89×10^{-3}<br>8.40×10^{-3}<br>1.92×10^{-2}	4.74×10^{-1}	0.9978
**1c**	1×10^{-3}<br>2×10^{-3}<br>4×10^{-3}<br>2×10^{-2}<br>4×10^{-2}	1.31×10^{-5}<br>2.01×10^{-5}<br>5.16×10^{-5}<br>1.88×10^{-4}<br>4.25×10^{-4}	1.04×10^{-2}	0.9976
**1d**	1×10^{-2}<br>2×10^{-2}<br>4×10^{-2}<br>6×10^{-2}<br>8×10^{-2}	3.32×10^{-4}<br>7.66×10^{-4}<br>1.56×10^{-3}<br>2.30×10^{-3}<br>2.92×10^{-3}	3.74×10^{-2}	0.9992
**1e**	1×10^{-1}<br>2×10^{-1}<br>4×10^{-1}<br>5×10^{-1}<br>6×10^{-1}	7.60×10^{-4}<br>2.05×10^{-3}<br>3.84×10^{-3}<br>5.06×10^{-3}<br>5.98×10^{-3}	1.03×10^{-2}	0.9989

Table S 4: The k_{obds} and k_1 values for the reactions of 2-methoxy-3-X-5-nitrothiophene **1a-e** with pyrrolidine in 40 % methanol - 60 % acetonitrile at 20 °C.

Thiophene	[Pyrrolidine] mol L^{-1}	k_{obds} s^{-1}	k_1 mol^{-1} L s^{-1}	Correlation coefficient
**1a**	6×10^{-4}	3.52×10^{-3}	4.96	0.9996
	7×10^{-4}	3.98×10^{-3}		
	8×10^{-4}	4.56×10^{-3}		
	9×10^{-3}	4.96×10^{-3}		
	1×10^{-3}	5.65×10^{-3}		
**1b**	1×10^{-2}	7.41×10^{-3}	7.41×10^{-1}	0.9900
	2×10^{-2}	1.18×10^{-2}		
	4×10^{-2}	2.96×10^{-2}		
	6×10^{-2}	4.84×10^{-2}		
	8×10^{-2}	5.57×10^{-2}		
**1c**	1×10^{-2}	1.41×10^{-3}	1.51×10^{-1}	0.9963
	2×10^{-2}	3.02×10^{-3}		
	4×10^{-2}	6.84×10^{-3}		
	6×10^{-2}	9.06×10^{-3}		
	8×10^{-2}	1.21×10^{-2}		
**1d**	1×10^{-2}	5.12×10^{-4}	5.15×10^{-2}	0.9997
	2×10^{-2}	1.02×10^{-3}		
	4×10^{-2}	2.13×10^{-3}		
	6×10^{-2}	3.12×10^{-3}		
	8×10^{-2}	4.10×10^{-3}		
**1e**	1×10^{-2}	1.65×10^{-3}	1.72×10^{-2}	0.9987
	2×10^{-2}	3.55×10^{-3}		
	2.5×10^{-2}	4.45×10^{-3}		
	3×10^{-2}	5.12×10^{-3}		
	3.5×10^{-2}	6.12×10^{-3}		
	4×10^{-2}	6.82×10^{-3}		

Table S 5: The k_{obds} and k_1 values for the reactions of 2-methoxy-3-X-5-nitrothiophene **1a-e** with pyrrolidine in 20 % methanol - 80 % acetonitrile at 20 °C.

Thiophene	[Pyrrolidine] mol L^{-1}	k_{obds} s^{-1}	k_1 mol^{-1} L s^{-1}	Correlation coefficient
**1a**	6 x 10^{-4}	5.61 x 10^{-3}	10.80	0.9998
	7 x 10^{-4}	6.73 x 10^{-3}		
	8 x 10^{-4}	7.75 x 10^{-3}		
	9 x 10^{-4}	8.90 x 10^{-3}		
	10 x 10^{-4}	9.92 x 10^{-3}		
**1b**	1 x 10^{-2}	1.28 x 10^{-2}	1.48	0.9945
	3 x 10^{-2}	4.44 x 10^{-2}		
	5 x 10^{-2}	7.40 x 10^{-2}		
	7 x 10^{-2}	9.36 x 10^{-2}		
	9 x 10^{-2}	1.36 x 10^{-1}		
**1c**	1 x 10^{-2}	2.41 x 10^{-3}	2.88 x 10^{-1}	0.9952
	3 x 10^{-2}	8.64 x 10^{-3}		
	5 x 10^{-2}	1.54 x 10^{-2}		
	7 x 10^{-2}	2.17 x 10^{-2}		
	9 x 10^{-2}	2.49 x 10^{-1}		
**1d**	1 x 10^{-2}	8.53 x 10^{-4}	9.53 x 10^{-2}	0.9994
	2 x 10^{-2}	1.91 x 10^{-3}		
	4 x 10^{-2}	3.68 x 10^{-3}		
	5 x 10^{-2}	4.65 x 10^{-3}		
	6 x 10^{-2}	5.70 x 10^{-3}		
**1e**	1 x 10^{-1}	3.20 x 10^{-3}	3.09 x 10^{-2}	0.9989
	2 x 10^{-1}	5.86 x 10^{-3}		
	3 x 10^{-1}	7.57 x 10^{-3}		
	4 x 10^{-1}	1.27 x 10^{-2}		
	5 x 10^{-1}	1.55 x 10^{-2}		
	6 x 10^{-1}	1.80 x 10^{-2}		

Table S 6: The k_{obds} and k_1 values for the reactions of 2-methoxy-3-X-5-nitrothiophene **1b-e** with pyrrolidine in pure acetonitrile at 20 °C

Thiophene	[Pyrrolidine] mol L^{-1}	k_{obds} s^{-1}	k_1 mol^{-1} L s^{-1}	Correlation coefficient
**1b**	1×10^{-3}	1.09×10^{-2}	10.9	0.9969
	2×10^{-3}	2.18×10^{-2}		
	4×10^{-3}	3.80×10^{-2}		
	6×10^{-3}	6.54×10^{-2}		
	8×10^{-3}	8.68×10^{-2}		
**1c**	2×10^{-3}	3.82×10^{-3}	1.91	0.9904
	4×10^{-3}	6.64×10^{-3}		
	6×10^{-3}	1.14×10^{-2}		
	8×10^{-3}	1.63×10^{-2}		
	10×10^{-3}	1.81×10^{-2}		
**1d**	6×10^{-3}	3.82×10^{-3}	8.14×10^{-1}	0.9990
	8×10^{-3}	6.12×10^{-3}		
	10×10^{-3}	8.06×10^{-3}		
	20×10^{-3}	1.49×10^{-2}		
	40×10^{-3}	3.20×10^{-2}		
**1e**	2×10^{-2}	7.00×10^{-3}	3.32×10^{-1}	0.9981
	4×10^{-2}	1.36×10^{-2}		
	6×10^{-2}	2.04×10^{-2}		
	8×10^{-2}	2.81×10^{-2}		
	10×10^{-2}	3.30×10^{-2}		

Table S 7: Values of second-order rate constants extrapolated from data of this work (see Figure 4) for the S_NAr reactions of 2-methoxy-3-X-5-nitrothiophenes **1a-e** with pyrrolidine in CH_3CN/CH_3OH mixtures containing 9, 33, 50 and 67% CH_3CN at 20 °C.

CH_3CN volume %	N_1 [b]	k_1 (L mol^{-1} s^{-1})				
		**1a** X = NO_2	**1b** X = CN	**1c** X = $COCH_3$	**1d** X = CO_2CH_3	**1e** X = $CONH_2$
9	7.45	1.40	1.96 x 10^{-1}	3.98 x 10^{-2}	1.38 x 10^{-2}	5.78 x 10^{-3}
33	7.01	2.58	3.66 x 10^{-1}	7.32 x 10^{-2}	2.52 x 10^{-2}	9.54 x 10^{-3}
50	6.67	4.15	5.93 x 10^{-1}	1.17 x 10^{-1}	4.02 x 10^{-2}	1.40 x 10^{-2}
67	6.38	6.22	8.95 x 10^{-1}	1.75 x 10^{-1}	6.00 x 10^{-2}	1.96 x 10^{-2}

[a]Calculated at 20 °C from Figures 4. [b]From ref. 1.

Interpolating solvent Nucleophilicity N_1

Minegishi et al. measured the solvent nucleophilicity N_1 for different methanol/acetonitrile ratios, reported in Table S 8.[1]

Table S 8: Solvent nucleophilicity N1 for different methanol/acetonitile ratios, from Ref 1.

CH_3CN volume %	0	9	20	33	50	67	80	90
N1	7.54	7.45	7.20	7.01	6.67	6.38	6.04	5.55

It appears that N_1 depends linearly on the volume amount of acetonitrile between 0% and 80% (R^2 = 0.9947):
N_1 = -0.0186 (%CH_3CN) + 7.5881

From this equation, we could interpolate N_1 for %CH_3CN = 40 and 60%: N_1(40%) = 6.84 ± 0.02 and N_1(60%) = 6.47 ± 0.03.

Using acetonitrile molar fractions

While most experiments used acetonitrile volume% amounts, it is also usual to characterize a mixture using molar fractions:

$$x(CH_3CN) = \frac{n(CH_3CN)}{n(CH_3CN) + n(CH_3OH)}$$

The number of acetonitrile and methanol molecules are deduced from the volumes by using the solvents volumic masses: $\rho(CH_3CN)$ = 786 g mol^{-1} and $\rho(CH_3OH)$ = 792 g mol^{-1}, and their molar masses: $M(CH_3CN)$ = 41.05 g mol^{-1} and $M(CH_3OH)$ = 32.04 g mol^{-1}.

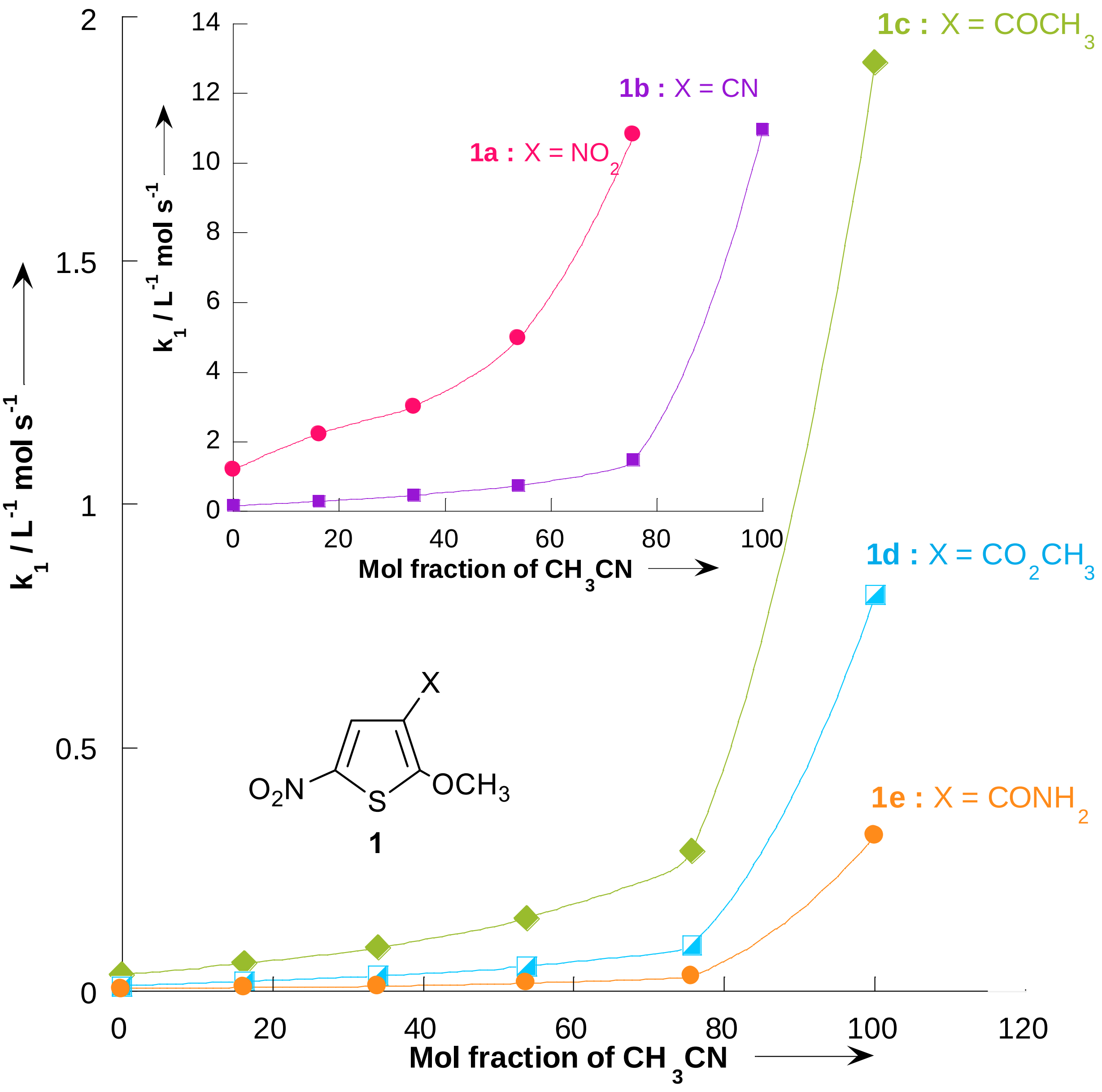

Figure S 1: Plots of the second-order rate constants for the reactions of pyrrolidine with the 2-methoxy-3-X-5-nitrothiophenes 1a-e against molar fraction of CH_3CN at 20 °C. Data from **Table S 9**.

Table S 9: Nucleophilicity parameter *N*, s for pyrrolidine and molar fraction of acetonitrile as a function of acetonitrile volume%

**% CH_3CN by volume**	0	9	20	40	60	80	91	100
N	15.72	15.97	16.02	16.27	16.51	17.02	17.73	18.32
s	0.64	0.64	0.64	0.62	0.63	0.65	0.62	0.61
**Mol fraction of CH_3CN**	0	7.129	16.205	34.024	53.762	75.604	88.717	100

Computational results

Computation details of Molecular Dynamic simulations.

The pyrrolidine molecule was placed in a cubic box containing approximately 1000 solvent molecules of methanol and acetonitrile. The initial box size was 41.4 Å. The concentration of pyrrolidine in these simulations is thus close to 2.3×10^{-2} mol/L. The number of solvent molecules was adjusted to reproduce the selected methanol/acetonitrile ratios used experimentally (Table S 10). Initial configurations were generated with PackMol[2]. One example for 60% of CH3CN in volume is shown in Figure S 2.

Table S 10: Number of solvent molecules used to simulate experimental solvent mixtures.

%vol ACN	0	9	20	40	60	80	91	100
Nb ACN	0	74	164	328	493	657	747	821
Nb MeOH	1060	964	848	636	424	212	95	0

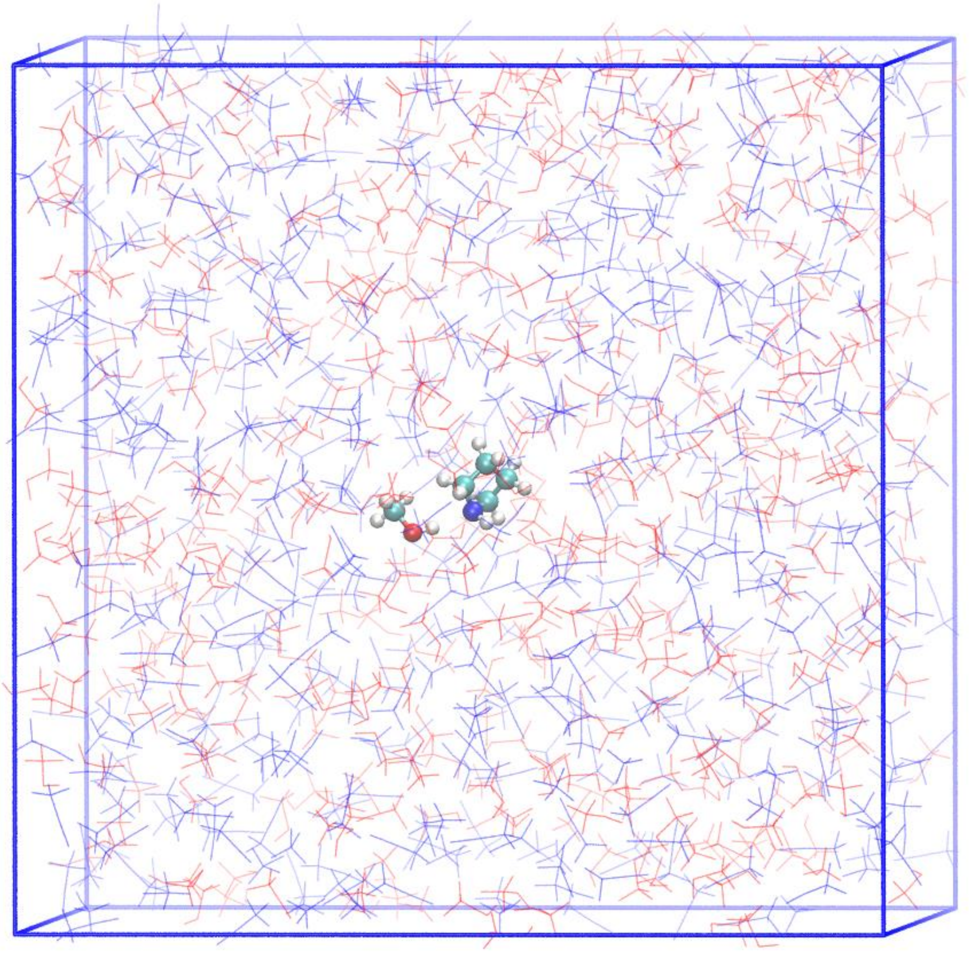

Figure S 2: Simulation box for 60% acetonitrile and 40% methanol in volume. The pyrrolidine and methanol in its first solvation layer are shown with ball&stick style. Acetonitrle molecules are shown in blue lines while methanol molecules are shown with red lines.

Methanol parameters were taken from the FFSB14 default set, Acetonitrile molecules are described with the six-site model of Grabuleda *et al.*[3] Pyrrolidine parameters were determined using Antechamber.

Bonds involving hydrogen atoms were constrained using the SHAKE algorithm during the MD simulations, and the timestep was set to 1fs. Each simulation was first minimized for 1000steps, then heated slowly to 300K in 1ns using the Berendsen thermostat, and then equilibrated in the NPT ensemble for 5ns, followed by 5ns of production run. Pressure was fixed to 1 bar using a relaxation time of 2ps. Temperature was fixed using a Langevin thermostat with a frequency of 1 ps^{-1}.

For each simulations, we extracted 5000 frames from the production run, separated by 1ps. We first analysed the radial distribution of methanol around the nitrogen of the pyrrolidine in pure methanol simulation to determine the width of the first solvation layer of pyrrolidine (Figure S 3).

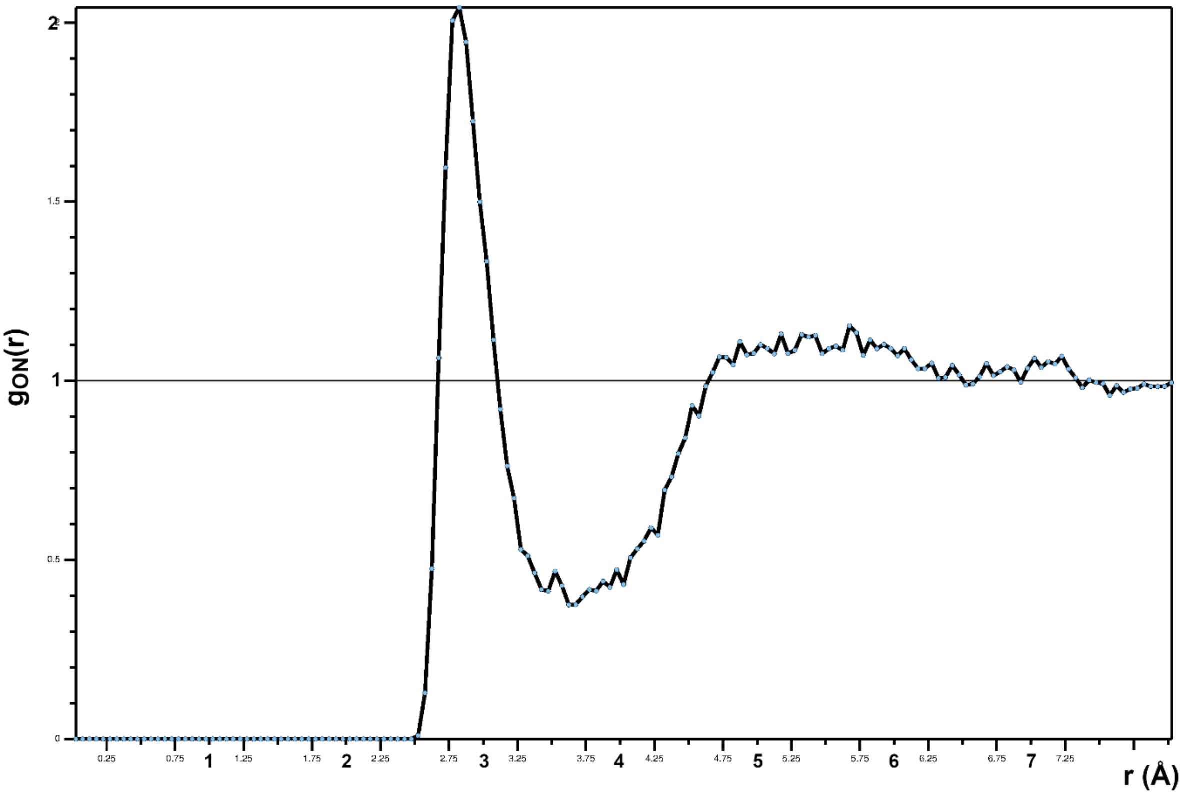

Figure S 3: Radial distribution function $g_{ON}(r)$ for nitrogen atom of the pyrrolidine and the oxygen atom of methanol performed during the last 5ns of MD simulation in pure methanol.

The minimum after the first peak lies between 3.5 and 4 Å approximately. To be on the safe side, we chose to define the first solvation shell as all molecules within 4 Å of the nitrogen atom of the pyrrolidine.

Then, for each frame we counted the number of methanol molecules in the first solvation shell of the pyrrolidine molecules (Table S 11).

Table S 11: Population and average number of methanol molecules (<n>) around pyrrolidine in various mixture solvent methanol-acetonitrile determined at 4 Å radius solvation cage.

% acetonitrile	0 CH_3OH	1 CH_3OH	2 CH_3OH	3 CH_3OH	4 CH_3OH	5 CH_3OH	<n>
**0%**	46	696	2673	1293	270	22	2.2224
**9%**	92	1015	2612	1083	179	19	2.06
**20%**	166	1372	2396	904	151	11	1.907
**40%**	416	1944	2067	506	62	5	1.5738
**60%**	633	2633	1456	244	31	3	1.2832
**80%**	1560	2649	724	67	0	0	0.8596
**91%**	2560	2158	266	15	1	0	0.5478
**100%**	5000	0	0	0	0	0	0

From these numbers, we could compute the probability P(n) that the pyrrolidine is surrounded by n methanol molecule for a given composition of the solvent methanol-acetonitrile (Table S 12). As expected, in simulations with more than 50% methanol, the pyrrolidine molecule is solvated by two explicit methanol molecules: one giving a H-bond to the nitrogen lone pair, and one accepting a H-bond from the N-H bond (Figure S 4). Then, for a given composition, the actual pyrrolidine nucleophilicity is the weighted average of the nucleophilicities computed for a given number of explicit methanol molecules:

$$\omega^{-1} = \frac{1}{\omega} = \sum_{n=0}^{n=5} P(n) \times \omega^{-1}(n)$$

Table S 12: Probability P(n) of finding n methanol molecule in the first solvation layer of pyrrolidine for different methanol-acetonitrile composition.

%CH_3CN (volume)	0 CH3OH	1 CH_3OH	2 CH_3OH	3 CH_3OH	4 CH_3OH	5 CH_3OH
**0%**	0.0092	0.1392	0.5346	0.2586	0.054	0.0044
**9%**	0.0184	0.203	0.5224	0.2166	0.0358	0.0038
**20%**	0.0332	0.2744	0.4792	0.1808	0.0302	0.0022
**40%**	0.0832	0.3888	0.4134	0.1012	0.0124	0.001
**60%**	0.1266	0.5266	0.2912	0.0488	0.0062	0.0006
**80%**	0.312	0.5298	0.1448	0.0134	0	0
**91%**	0.512	0.4316	0.0532	0.003	0.0002	0
**100%**	1	0	0	0	0	0

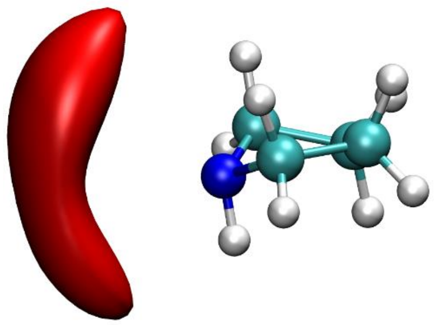

Figure S 4: Probability of presence of the methanol oxygen around the pyrrolidine.

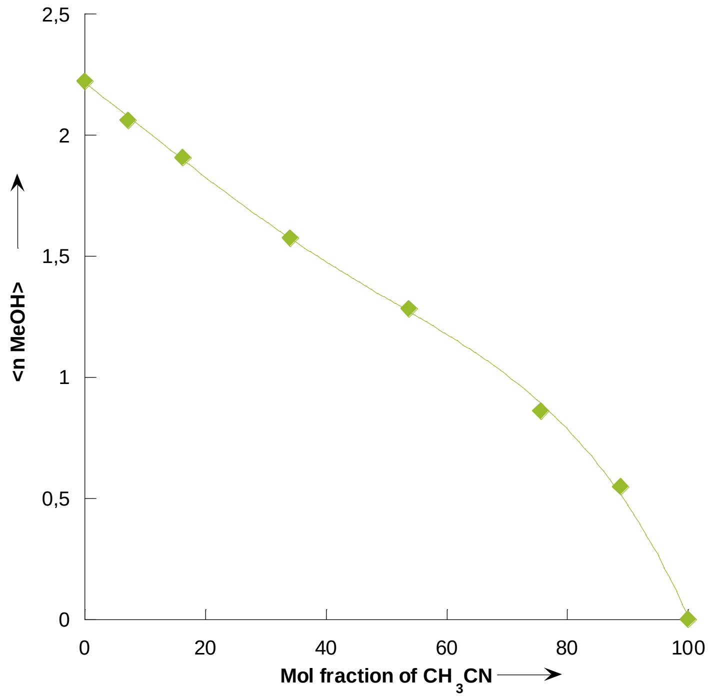

Figure S 5: Average methanol molecules in the first solvation shell of pyrrolidine as a function of the molar fraction of acetonitrile.

To estimate the uncertainty of the computed average nucleophilicities the simulation with 0% and 91% of acetonitrile were run for an additional 10ns. The average nucleophilicities were then computed for three 5ns interval (from 5ns to 10ns, from 10ns to 15ns and from 15ns to 20ns): for both mixture, we found that the uncertainty for a 95% confidence is 0.15 only.

As the pyrrolidine concentration in our simulations is close to the highest experimental concentrations (see Tables S1 to S6), we ran a simulation with a concentration of 7.9×10^{-3} mol/L to check the influence of the pyrrolidine concentration on the distribution of methanol. Results are gathered in Table S 13 and show that the distribution of methanol in the first solvation layer is quite similar for both simulations.

Table S 13: Probability P(n) of finding n methanol molecule in the first solvation layer of pyrrolidine for different concentration in pyrrolidine.

[Pyr] (mol/L)	0 CH3OH	1 CH_3OH	2 CH_3OH	3 CH_3OH	4 CH_3OH
2.3×10^{-2}(a)	0.5120	0.4316	0.0532	0.0030	0.0002
7.9×10^{-3}(b)	0.5084	0.4090	0.0734	0.0076	0.0002

(a) 1 pyrrolidine molecule in a cubic box of 41.7 Å length (b) 1 pyrrolidine molecule in a cubic box of 59.5 Å length.

Last, the robustness of this protocol was checked for the case of 40% acetonitrile: we extracted the pyrrolidine and the methanol molecules in its first solvation layer for the last 3000 frames of the MD simulation. The nucleophilic parameter ω^{-1} was then computed for each frame using a single point calculation at the B3LYP-D3/6-311++G (d,p) level. This procedure gave $\omega^{-1} = 28.03 \pm 0.05$, in very good agreement with the valued obtained by re-sampling protocol: $\omega^{-1} = 28.33$.

Influence of the PCM parameters

The influence of PCM parameters on the nucleophilic parameters ω^{-1} was tested by computing ω^{-1} in a hybrid solvent for which the dielectric constant was linearly interpolated between the value of acetonitrile (ε_r= 35.688) and methanol (ε_r = 32.613). Results are gathered in Table S 14 and confirm that explicit solvation has to be taken into account.

Table S 14: Nucleophilic parameters ω^{-1} of pyrrolidine surrounded by a number n of methanol molecule measured by B3LYP / 6-311 ++ G (d, p) calculation

% acetonitrile	ε	ω^{-1} 0 CH_3OH
**0%**	32.613	30.2
**9%**	32.889	30.2
**20%**	33.227	30.2
**40%**	33.842	30.2
**50%**	34.150	30.2
**60%**	34.457	30.1
**80%**	35.072	30.2
**91%**	35.380	30.2
**100%**	35.688	30.2

References

[1] Minegishi S, Kobayashi S, Mayr H. *Solvent nucleophilicity*. J. Am. Chem. Soc. **2004**; *126*, 5174-5181.
[2] Martínez L., Andrade R., Birgin E. G., Martínez J. M. *Packmol: A package for building initial configurations for molecular dynamics simulations.* J. Comput. Chem. **2009**, *30(13)*,2157-2164.
[3] Grabuleda X, Jaime C, Kollman PA J. *Molecular dynamics simulation studies of liquid acetonitrile: New six-site model* Comp. Chem. **2000**,21,901
